# Validity and Reliability of Self-Reported Prevalent and Incident Cardiovascular Disease Among Asian Adults

**DOI:** 10.3390/jcdd11110350

**Published:** 2024-11-01

**Authors:** Charumathi Sabanayagam, Feng He, Miao Li Chee, Ching-Yu Cheng

**Affiliations:** 1 Singapore Eye Research Institute, Singapore National Eye Centre, Singapore 169856, Singapore; feng.he@u.nus.edu (F.H.); chee.miao.li@seri.com.sg (M.L.C.); chingyu.cheng@duke-nus.edu.sg (C.-Y.C.); 2 Ophthalmology and Visual Sciences Academic Clinical Program, Duke-NUS Medical School, Singapore 169856, Singapore; 3 Department of Ophthalmology, Yong Loo Lin School of Medicine, National University of Singapore, Singapore 119228, Singapore

**Keywords:** accuracy, acute myocardial infarction, incident CVD, prevalent CVD, self-report, stroke

## Abstract

Cardiovascular disease (CVD) is the leading cause of death in Asians. We aimed to examine the validity and reliability of self-reported (SR) CVD in 6762 Chinese, Malay, and Indian adults aged 40–80 years who attended the baseline (November 2004) and 6-year follow-up visit (2011–2017) of a population-based cohort study in Singapore. CVD was defined based on the presence of existing (prevalent) or new onset (incident) cases of acute myocardial infarction (AMI) or stroke. The validity of SR-CVD was assessed by comparing it against diagnosed CVD using sensitivity and specificity. The reliability of SR-CVD was evaluated by calculating the percentage of positive agreement between baseline and follow-up visits. The sensitivity and specificity of SR-CVD were 62.7% and 93.8% for prevalent SR-CVD and 50.9% and 98.5% for incident SR-CVD. The negative predictive value (NPV) was 98.1% for both prevalent and incident SR-CVD. The reliability of positive self-reports between the baseline and follow-up was substantial, at 85%. The excellent specificity and NPV of SR-CVD suggest that it could serve as a valuable tool for excluding AMI and stroke. However, its moderate sensitivity suggests that positive SR-CVD reports should prompt further clinical evaluation to prevent potential false positives.

## 1. Introduction

Cardiovascular disease (CVD), encompassing coronary artery disease and stroke, is the leading cause of death and disability worldwide and, to be particular, in Asians [1]. A recent review showed that of the 18.6 million global CVD deaths, over half of them occurred in Asian countries [2]. Thus, identifying the magnitude of CVD and obtaining reliable estimates of CVD in Asian populations is important for planning the allocation of healthcare resources and devising intervention strategies to prevent the progression of CVD to disability and death. CVD is often defined using self-reported (SR) information in large-scale epidemiological studies, as assessment by SR history is cost-effective as well as efficient, where information is collected based on just questionnaires.

Several studies have examined the reliability (agreement) or validity (accuracy) of prevalent SR-CVD. These studies conducted in predominantly white populations have revealed varying levels of agreement [3,4] or accuracy [4,5,6,7,8,9]. Specifically, the positive predictive value (PPV) for myocardial infarction (MI) and stroke in these studies has exhibited a wide range, spanning from 33% to 84% [8]. It is important to note that there is a paucity of studies validating the accuracy of incident SR-CVD cases [5,10]. This gap in research is particularly pronounced within Asian populations, which are considered high-risk groups for CVD. Surprisingly, only one Japanese study assessed the accuracy of incident stroke and MI in a large cohort of middle-aged adults who reported a positive history [10]. This highlights the need for further investigation in assessing the accuracy of SR-CVD (both positive as well as negative history) within diverse populations, especially those with distinct risk profiles, such as Asian individuals, which will be crucial for enhancing the reliability of self-reported data in healthcare studies. In the current study, we aimed to (1) assess the validity and reliability of prevalent SR-CVD and (2) evaluate the validity of incident SR-CVD by comparing it to diagnosed CVD recorded in the National Disease Registry in Singapore. Additionally, we evaluated whether there were any differences in the validity of SR-CVD based on age, gender, ethnicity, or education level.

## 2. Materials and Methods

The Singapore Epidemiology of Eye Diseases (SEED) study provided an opportunity to investigate the validity of SR-CVD and examine if there were inter-ethnic differences in reporting. SEED is a population-based cohort study of 10,023 Chinese, Malay, and Indian participants who participated in the baseline (2004–2011) [11] and 6-year follow-up visits (2011–2017) [12]. We used baseline data to identify participants with pre-existing SR-CVD and excluded these cases to calculate incident CVD events. 

### 2.1. Assessment of Self-Reported and Definitive CVD

Participants were classified as having SR-CVD if they answered, ‘Yes’ to either of the following questions: “Has a doctor advised you that you have heart attack?” or “Has a doctor advised you that you have stroke” at both the baseline and follow-up visits. To increase validity, these questions were followed by questions on hospitalization: “Were you admitted to hospital?” “To which hospital were you admitted?” “What is your treatment history supplementary to a heart attack?” “Have you undergone a bypass, angioplasty, pacemaker, valvular replacement or other type of treatment?” Definitive diagnoses of CVD events including AMI and stroke were obtained by linking baseline data to the National Registry of Diseases Office (NRDO) and the National Death Index, which contains records of CVD events deaths due to these events from January 2007 until 31 December 2019. Although the SEED study included a question on the history of angina, since information on angina was not collected at the NRDO, we defined SR-CVD in this study based on the history of stroke and AMI. We included only participants who attended both the baseline and 6-year follow-up visits and had registry linkage data available, excluding those who died from CVD in the registry before the SEED Visit 2. In total, 6762 participants who attended both visits were included for the prevalent CVD analysis. After excluding those with prevalent SR-CVD at baseline, 6309 participants were included for the incident CVD analysis.

### 2.2. Definitions

Prevalent CVD: Prevalent CVD was classified into prevalent SR-CVD and prevalent diagnosed CVD based on participants’ responses and registry data. Participants who answered yes to the questions on previous AMI and stroke in Visit 2 were classified as having prevalent SR-CVD. For AMI, the questionnaire also included treatment for heart attack (coronary artery bypass graft [CABG] or angioplasty) and whether they had been admitted to the hospital for treatment. In the registry, participants coded (International Classifications of Diseases [ICD]) as having had AMI or stroke before Visit 2 were considered to have prevalent diagnosed CVD. ICD codes were used to identify AMI and stroke cases. For AMI, ICD-9 code 410 was used prior to 2012, while ICD-10 Australian Modification codes 121 to 122 were used from 2012 onwards. For stroke, ICD-9 codes 430 to 437 (excluding 432.1 and 435) were used prior to 2012, and ICD-10 Australian Modification codes 160 to 168 (excluding 162.0 and 162.1) were used for stroke cases diagnosed from 2012 onwards.

Incident CVD: Similarly to prevalent CVD, incident CVD was classified into incident SR-CVD and incident diagnosed CVD based on participants’ responses and registry data. Incident SR-CVD was defined as those without pre-existing CVD in Visit 1 but reported a CVD event in Visit 2. Incident diagnosed CVD was defined as new CVD events occurring after the baseline visit but before Visit 2 in those without pre-existing CVD as identified by self-report.

The reliability of prevalent SR-CVD was assessed based on SR-CVD data from both the baseline and 6-year follow-up visits. Reliability was defined as the consistent reporting of a history of previously diagnosed CVD both at both the baseline and 6-year follow-up visits in the SEED study.

### 2.3. Assessment of Covariates

Information on participants’ demographic characteristics (age, sex, ethnicity), lifestyle (cigarette smoking and alcohol consumption), and education status (highest education level obtained) was collected using a standardized questionnaire. The physical examination included height, weight, and blood pressure measurements. Laboratory examinations included random blood glucose, HbA1c %, serum cholesterol levels (total, high-density lipoprotein [HDL] and low-density lipoprotein [LDL]). Education was classified into primary/below (≤6 years) or secondary/above (>6 years). The body mass index (BMI) was calculated as weight in kilograms divided by height in meters squared (kg/m^2^). BMI categories included underweight (<18.5 kg/m^2^), normal (18.5–24.9 kg/m^2^), overweight (25–29.9 kg/m^2^), and obese (≥30 kg/m^2^). The classification of current smoking, alcohol consumption, diabetes, hypertension, and dyslipidemia have been described before [12].

### 2.4. Statistical Analysis

We compared the characteristics of participants by prevalent diagnosed CVD outcome recorded in the Registry by Chi-square tests or Student’s t-tests as appropriate for the variable. Reliability is typically assessed by both positive and negative self-reports; however, since the preliminary analysis indicated high specificity for SR-CVD, we estimated reliability by focusing solely on positive self-reports. This was conducted by calculating the percentage of positive agreement (pairwise agreement between baseline and the follow-up visit) among those who self-reported CVD at baseline. We assessed validity by comparing participants’ self-reports at the 6-year follow-up to diagnosed CVD, with validity metrics including sensitivity, specificity, positive predictive value (PPV), and negative predictive value (NPV) by comparing SR-CVD with diagnosed CVD for both prevalent and incident CVD outcomes. We repeated the same analysis stratified by age (<60 vs. ≥60 years), sex, ethnicity (Malays and Indians vs. Chinese), and education level (primary/below vs. secondary/above).

## 3. Results

**Figure 1:**
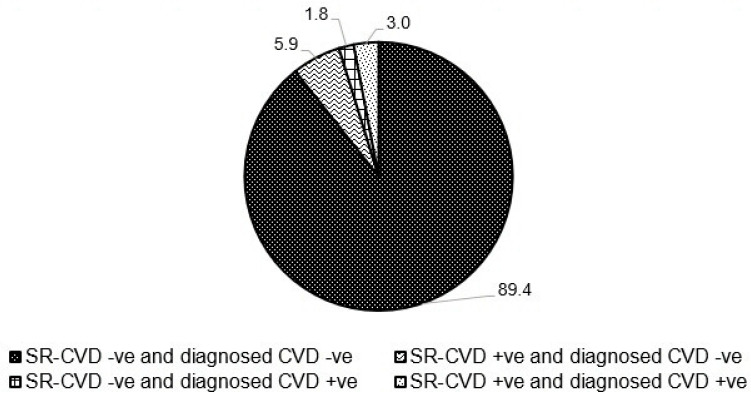
Accuracy of self-reported CVD compared to diagnosed CVD at baseline.

Table 1 shows the characteristics of participants by prevalent diagnosed CVD. Of the total 6762 participants, 4.7% had prevalent diagnosed CVD. Compared to those without diagnosed CVD, those with were more likely to be older, men, of Indian ethnicity, and primary/below educated. Additionally, they had a higher likelihood of being overweight or obese, current smokers, and had higher prevalence of hypertension, dyslipidemia, and diabetes. Furthermore, they exhibited lower levels of both total and HDL cholesterol (all *p* < 0.05). Figure 1 shows the proportion of participants with CVD based on self-report, diagnosed, or both criteria at baseline. Of the 6762 participants, 6046 (89.4%) were negative for prevalent CVD by both self-report and diagnosed; 397 (5.9%) had SR-CVD only; 119 (1.8%) had diagnosed CVD only; and 200 (3%) had prevalent CVD by both criteria. In other words, while 92.4% showed concordant results for prevalent CVD, 7.7% had discordant results. By CVD subtype, discordance was observed in 6% for AMI and 2.4% for stroke.

Table 2 presents the sensitivity, specificity, PPV, and NPV for both prevalent SR-CVD and incident SR-CVD. For prevalent SR-CVD, the sensitivity was 62.7% and the PPV was 33.5%. The specificity and NPV were notably high, at 93.8% and 98.1%. When stratified by AMI and stroke, all metrics were significantly higher for SR-stroke compared to SR-AMI. Within the SR-AMI group, sensitivity was particularly high for CABG at 82.4% compared to angioplasty at 53.2%. The specificity, PPV, and NPV were similar between the two treatment types.

For incident SR-CVD, we observed similar patterns as seen with prevalent SR-CVD. The sensitivity for total CVD was lower at 50.9%, but the specificity and NPV remained high at 98.5% and 98.1%. Interestingly, the PPV for incident SR-CVD was higher at 56.1% compared to 33.5% for prevalent SR-CVD. Similarly, within the SR-AMI group, sensitivity was high for CABG (75%) but was notably low for angioplasty (20%).

Validation metrics stratified by age, gender, ethnicity, and education level are presented in Table 3 (prevalent SR-CVD) and Table 4 (incident SR-CVD). For both prevalent and incident SR-CVD, sensitivity was lower among (1) ages ≥60 years (61.6%, 48.0%) compared to ages <60 years (64.1%, 54.2%); (2) women (58.9%, 48.6%) compared to men (64.3%, 51.9%); (3) the Chinese-ethnicity (60.5%, 33.3%) compared to Indians (64.1%, 51.6%) and Malays (62.6%, 59.1%); and (4) secondary/above education (61.2%, 50.0%) compared to primary/below education (63.6%, 51.6%).

In analyses evaluating the reliability of prevalent SR-CVD, we found substantial reliability with a positive agreement of 85% between baseline and follow-up visits. When comparing the reliability between AMI and stroke, the reliability was high for SR-stroke at 88.2% compared to 81.7% for SR-AMI.

## 4. Discussion

Our study involving a multi-ethnic Asian population demonstrated that prevalent SR-CVD and incident SR-CVD including AMI and stroke had high specificity and NPV when validated against diagnosed CVD. However, the sensitivity and PPV were modest. Between AMI and stroke, sensitivity was significantly higher for SR-stroke, while specificity remained similar for both. Within SR-AMI, CABG had higher sensitivity compared to angioplasty. In subgroup analyses, sensitivity for SR-CVD was higher for men compared to women, Malays and Indians compared to the Chinese ethnicity, adults aged <60 years compared to those aged ≥60 years, and those with primary/below education compared to secondary/above education level. We observed similar patterns in both cross-sectional analyses of prevalent SR-CVD as well as longitudinal analyses of incident SR-CVD. The reliability of prevalent SR-CVD was substantial, with 85% at two time points.

### 4.1. Prevalent SR-CVD

In the current study, the sensitivity and specificity for prevalent SR-CVD were 62.7% and 93.8% with the PPV and NPV at 33.5% and 98.1%. At baseline, 7.7% had discordant results for total CVD, while 6% and 2.4% had discordant results for AMI and stroke. For SR-AMI, the sensitivity and specificity were 56.5% and 95.2% with the PPV and NPV at 27.9% and 98.5%. Similarly to our findings, other studies have reported modest sensitivity for SR-AMI. Two previous reports from the US reported 60% sensitivity for SR-AMI [13,14]. In the Health and Retirement Study, a US-based matched case–control study using Medicare claims, the sensitivity of SR-AMI was higher at 67.8% and the PPV at 32.8% [15]. However, this study used claims data as the reference standard, which may be less accurate than medical records or registry data.

Our study found that SR-CVD was more effective for identifying stroke than AMI. For SR-stroke, sensitivity and specificity were 61.7% and 98.2%, consistent with the Health in Men Study in Australia, which reported 65% sensitivity and 96% specificity for SR-stroke based on hospital coded records [7]. A recent systematic review based on 10 studies using population-based reference standards across several countries, including North America, Australia, UK, and Japan, showed that the sensitivity of SR-stroke was variable, ranging from 36 to 98%, whereas specificity (96–99.6%) and NPV (88.2–99.9%) were consistently high [16]. Authors demonstrated that when the prevalence of stroke was low (<10%), false positive rates were higher (~1/3 to 2/3), affecting the PPV. In our study, the PPV of stroke and AMI were low at 37.4% and 27.9%, but the NPV exceeded 98% for both conditions. Choe et al. validated 208 patients with SR-stroke and 221 patients with SR-MI who participated in the Health Examinees Study (HEXA) in Korea by cross referencing with a review of medical records [17]. Authors reported a PPV of 51.4% for stroke and 32.6% for MI. Though their PPV was higher than ours, they did not validate those who did not report stroke or MI (controls). By not validating individuals who did not report stroke or MI, false negatives were not accounted for, which may have led to an overestimation of the prevalence and PPV of stroke and AMI.

### 4.2. Incident SR-CVD

In our study, incident SR-CVD demonstrated a sensitivity of 50.9% and a specificity of 98.5% when compared to diagnosed CVD. Three previous studies assessing the validity of SR-MI and SR-stroke reported higher sensitivity than the current study. Barr et al., using physician adjudication to validate incident SR-CVD in a sample of 276 participants from the AusDiab cohort, found that 188 were diagnosed with CVD (AMI, stroke, CABG, or PTCA), resulting in a sensitivity of 68.1% [5]. In the Greek EPIC cohort, the sensitivity of SR-MI and SR-stroke through medical record validation were 65% and 72% [18]. In the Japan Public Health Center study, SR-MI and SR-stroke, as validated through registry coding, were 82% and 72% among 91,186 participants completing the 10-year follow-up survey [10]. However, all three studies [5,10,18] focused only on participants who reported a positive history of CVD, so specificity and NPV were not assessed.

In the AusDiab Cohort, CABG had an accuracy of 100%, and in our study, we also observed relatively high sensitivity for CABG at 75%. We found the sensitivity for SR-CVD to be lower among adults aged ≥60 years compared to those under 60. Similarly, in the Health and Retirement Study conducted in the US, older adults (>75 years) were less likely to accurately report a heart attack than younger ones (62.7% versus 74.6%; *p* = 0.006) [15]. This suggests that older adults face more challenges in accurately recalling and reporting past CVD events.

In the current study, similar to prevalent SR-CVD, sensitivity for incident SR-stroke (53.2%) was higher than for incident SR-AMI (46.9%). In the longitudinal analysis of the Health in Men Study in Australia, incident SR-stroke demonstrated a sensitivity of 65% and a PPV of 52% with a specificity and NPV of 96% and 98% [7]. When stratified by AMI and stroke in our study, we observed discordant results in 2.3% and 1.2% of cases for incident AMI and stroke. In comparison, Tey et al. reported higher discordance rates of 12% for incident stroke and 16% for incident AMI in 937 octogenarians in New Zealand’s Life and Living to Advanced Age cohort study [4].

The lower sensitivity observed in the current study, particularly for incident CVD events, could have several plausible explanations. First, participants may not have accurately distinguished between AMI and unstable angina or stroke and transient ischemic attacks (TIAs), as well as other conditions such as heart failure. This potential lack of differentiation could have led to an over- or under-reporting of these conditions. In analyses evaluating all-cause mortality, we found that false positive SR-CVD cases had a higher all-cause mortality (26%) compared to true positives (19%), suggesting that those identified as false positive may have other cardiac or non-cardiac illnesses that they confused with AMI/stroke. Rosamond et al. showed that 40% of the false positive reporting for AMI was due to unstable angina [14]. In our study, since the registry had only hard outcomes like stroke and AMI, this limited our ability to verify whether individuals who self-reported AMI or stroke had other CVD conditions. Using a broader definition of CVD, including angina, TIA, heart failure, etc., as well as AMI and stroke, would have improved the sensitivity of SR-CVD. Second, study participants might have recalled their medical history inaccurately due to memory lapses, inadvertently reporting events or conditions that did not occur, or they may have over-reported minor CVD conditions due to health anxiety. Third, it is possible that participants might have sought medical care in hospitals outside of Singapore, leading to these events being not captured in the registry. 

Our study has several notable strengths. First, it encompasses a large population-based sample that included Chinese, Malay, and Indian populations, the three major ethnic groups in Asia. Second, the reference standards used in the study utilized data from a nationwide registry, encompassing all hospitalized cases of stroke and AMI within public hospitals in Singapore. This robust reference standard further strengthens the validity and credibility of our study findings. Our study has some limitations. We included only participants who attended both the baseline and follow-up visits of the SEED study, excluding those who died of CVD between these visits, which may have underestimated the sensitivity of SR-CVD. In addition, our study relied on diagnostic codes from a nationwide registry, employing standardized definitions and case report forms for consistency in data collection, along with rigorous quality assurance procedures. While this registry provides a comprehensive dataset of hospitalized stroke and AMI cases in public hospitals, the exclusive use of diagnostic codes may limit the accuracy of specific CVD case identification. Incorporating procedure and medication codes could improve case identification accuracy. These limitations should be considered when interpreting our findings.

## 5. Conclusions

In conclusion, SR-CVD including SR-AMI and SR-stroke demonstrated strong performance in terms of specificity and NPV for both prevalent and incident CVD. These attributes suggest that SR-CVD could serve as a valuable tool for ruling out the presence of AMI and stroke. However, the modest sensitivity of both prevalent and incident SR-CVD suggests that individuals reporting a positive SR-CVD history should undergo additional clinical evaluation to confirm the diagnosis, thereby avoiding potential false positives. Expanding the definition of CVD to encompass conditions like angina, heart failure, and TIA may improve the validity of positive self-reports.

## Figures and Tables

**Table 1:** Characteristics of participants included in the validation study by prevalent diagnosed CVD.

Characteristics	All	Diagnosed CVD		*p*-Value
		Yes	No	
	(n = 6762)	(n = 319)	(n = 6443)	
Age, years	63.5 (9.6)	68.0 (10.1)	63.3 (9.5)	<0.001
Sex				
Women, %	3515 (52.0)	95 (29.8)	3420 (53.1)	<0.001
Men, %	3247 (48.0)	224 (70.2)	3023 (46.9)	
Ethnicity, %				<0.001
Chinese	2661 (39.4)	81 (25.4)	2580 (40.0)	
Malay	1901 (28.1)	107 (33.5)	1794 (27.8)	
Indian	2200 (32.5)	131 (41.1)	2069 (32.1)	
Education level *, %				<0.001
Primary/below	3682 (54.5)	220 (69.2)	3462 (53.8)	
Secondary/above	3075 (45.5)	98 (30.8)	2977 (46.2)	
BMI Category, * %				0.9
Underweight	266 (4.0)	12 (4.0)	254 (4.0)	
Normal	3113 (46.7)	134 (44.4)	2979 (46.8)	
Overweight	2313 (34.7)	109 (36.1)	2204 (34.7)	
Obese	970 (14.6)	47 (15.6)	923 (14.5)	
BMI, kg/m^2^	25.5 (4.7)	25.5 (4.8)	25.5 (4.7)	0.8
Current smoking, %	873 (12.9)	76 (23.9)	797 (12.4)	<0.001
Alcohol consumption, %	530 (7.9)	26 (8.3)	504 (7.9)	0.9
Diabetes, yes, %	2091 (30.9)	154 (48.4)	1937 (30.1)	<0.001
Hypertension, yes, %	4698 (69.8)	287 (90.5)	4411 (68.7)	<0.001
Dyslipidemia, yes, %	3802 (60.5)	232 (81.7)	3570 (59.5)	<0.001
Total cholesterol, mmol/L	5.4 (1.2)	4.6 (1.2)	5.4 (1.2)	<0.001
HDL cholesterol, mmol/L	1.3 (0.3)	1.2 (0.3)	1.3 (0.3)	<0.001

Abbreviations: BMI: body mass index; HDL: high-density lipoprotein. * Missing numbers for education level (n = 5) and BMI (n = 10).

**Table 2:** Validation of prevalent SR-CVD at baseline and incident SR- CVD between baseline and 6-year follow-up visit.

Outcome CVD Diagnoses	No. of Cases (Self-Reported)	No. of Cases (Diagnosed)	Sensitivity (95% CI), %	Specificity (95% CI), %	PPV (95% CI), %	NPV (95% CI), %
Prevalent CVD						
Total CVD	597	319	62.7 (57.1–68.0)	93.8 (93.2–94.4)	33.5 (29.7–37.4)	98.1 (97.7–98.4)
AMI	434	214	56.5 (49.6–63.3)	95.2 (94.7–95.7)	27.9 (23.7–32.4)	98.5 (98.2–98.8)
Stroke	190	115	61.7 (52.2–70.6)	98.2 (97.9–98.5)	37.4 (30.5–44.7)	99.3 (99.1–99.5)
Supplementary to AMI						
CABG	145	34	82.4 (65.5–93.2)	98.3 (97.9–98.6)	19.3 (13.2–26.7)	99.9 (99.8–100.0)
Angioplasty	173	62	53.2 (40.1–66.0)	97.9 (97.5–98.2)	19.1 (13.5–25.7)	99.6 (99.4–99.7)
Incident CVD						
Total CVD	212	234	50.9 (44.3–57.4)	98.5 (98.1–98.8)	56.1 (49.2–62.9)	98.1 (97.7–98.4)
AMI	142	162	46.9 (39.0–54.9)	98.9 (98.6–99.2)	53.5 (45.0–61.9)	98.6 (98.3–98.9)
Stroke	76	77	53.2 (41.5–64.7)	99.4 (99.2–99.6)	53.9 (42.1–65.5)	99.4 (99.2–99.6)
Supplementary to AMI						
CABG	29	4	75.0 (19.4–99.4)	99.6 (99.4–99.7)	10.3 (2.2–27.4)	100.0 (99.9–100.0)
Angioplasty	53	20	20.0 (5.7–43.7)	99.2 (99.0–99.4)	7.5 (2.1–18.2)	99.7 (99.6–99.9)

**Table 3:** Validation of prevalent SR-CVD in subgroups.

Participant Subgroups	No. of Cases (Self-Reported)	No. of Cases (Diagnosed)	Sensitivity (95% CI), %	Specificity (95% CI), %	PPV (95% CI), %	NPV (95% CI), %
Age, years						
Age < 60	220	142	64.1 (55.6–72.0)	96.8 (96.2–97.3)	41.4 (34.8–48.2)	98.7 (98.3–99.0)
Age ≥ 60	377	177	61.6 (54.0–68.8)	88.7 (87.4–90.0)	28.9 (24.4–33.8)	96.9 (96.1–97.6)
Gender						
Women	182	95	58.9 (48.4–68.9)	96.3 (95.6–96.9)	30.8 (24.2–38.0)	98.8 (98.4–99.2)
Men	415	224	64.3 (57.6–70.6)	91.0 (90.0–92.0)	34.7 (30.1–39.5)	97.2 (96.5–97.8)
Ethnicity						
Chinese	152	81	60.5 (49.0–71.2)	96.0 (95.2–96.7)	32.2 (24.9–40.3)	98.7 (98.2–99.1)
Malay	186	107	62.6 (52.7–71.8)	93.4 (92.1–94.5)	36.0 (29.1–43.4)	97.7 (96.8–98.3)
Indian	259	131	64.1 (55.3–72.3)	91.5 (90.3–92.7)	32.4 (26.8–38.5)	97.6 (96.8–98.2)
Education level						
Primary/below	397	220	63.6 (56.9–70.0)	92.6 (91.7–93.4)	35.3 (30.6–40.2)	97.6 (97.0–98.1)
Secondary/above	200	98	61.2 (50.8–0.9)	95.3 (94.5–6.0)	30.0 (23.7–36.9)	98.7 (98.2–99.1)

**Table 4:** Validation of incident SR-CVD in subgroups.

Participant Subgroups	No. of Cases (Self-Reported)	No. of Cases (Diagnosed)	Sensitivity (95% CI), %	Specificity (95% CI), %	PPV (95% CI), %	NPV (95% CI), %
Age, years						
Age < 60	104	107	54.2 (44.3–63.9)	98.8 (98.5–99.1)	55.8 (45.7–65.5)	98.8 (98.4–99.1)
Age ≥ 60	108	127	48.0 (39.1–57.1)	97.8 (97.1–98.4)	56.5 (46.6–66.0)	96.9 (96.1–97.6)
Gender						
Women	71	72	48.6 (36.7–60.7)	98.9 (98.5–99.2)	49.3 (37.2–61.4)	98.9 (98.5–99.2)
Men	141	162	51.9 (43.9–59.8)	97.9 (97.3–98.4)	59.6 (51.0–67.7)	97.2 (96.5–97.8)
Ethnicity						
Chinese	32	48	33.3 (20.4–48.4)	99.4 (99.0–99.6)	50.0 (31.9–68.1)	98.7 (98.2–99.1)
Malay	113	93	59.1 (48.5–69.2)	96.6 (95.6–97.4)	48.7 (39.2–58.3)	97.7 (96.9–98.4)
Indian	67	93	51.6 (41.0–62.1)	99.0 (98.4–99.4)	71.6 (59.3–82.0)	97.7 (96.9–98.3)
Education level						
Primary/below	145	159	51.6 (43.5–59.6)	98.0 (97.5–98.5)	56.6 (48.1–64.8)	97.6 (97.0–98.1)
Secondary/above	67	74	50.0 (38.1–61.9)	98.9 (98.5–99.3)	55.2 (42.6–67.4)	98.7 (98.2–99.1)

## Data Availability

As the study involves human participants and cardiovascular disease outcomes, restricted data are used. Due to data access limitations, even with a formal request, the raw data cannot be made available. Analyses are conducted directly within the disease registry, which provides only aggregate output to the research team. All relevant aggregate data are already presented in the study tables.
